# A first-generation genome-wide map of correlated DNA methylation demonstrates highly coordinated and tissue-independent clustering across regulatory regions

**DOI:** 10.21203/rs.3.rs-2852818/v1

**Published:** 2023-05-15

**Authors:** Aarti Jajoo, Owen Hirschi, Katharina Schulze, Yongtao Guan, Neil A. Hanchard

**Affiliations:** 1 Department of Molecular and Human Genetics, Baylor College of Medicine, Houston, TX, USA.; 2 USDA/ARS/Children’s Nutrition Research Center, Baylor College of Medicine, Houston, TX, USA.; 3 Center for Precision Health Research, National Human Genome Research Institute, National Institutes of Health, Bethesda, MD, USA

**Keywords:** epigenetics, methylation, methylation patterns

## Abstract

Genome-wide DNA methylation studies have typically focused on quantitative assessments of CpG methylation at individual loci. Although methylation states at nearby CpG sites are known to be highly correlated, suggestive of an underlying coordinated regulatory network, the extent and consistency of inter-CpG methylation correlation across the genome, including variation between individuals, disease states, and tissues, remains unknown. Here, we leverage image conversion of correlation matrices to identify correlated methylation units (CMUs) across the genome, describe their variation across tissues, and annotate their regulatory potential using 35 public Illumina BeadChip datasets spanning more than 12,000 individuals and 26 different tissues. We identified a median of 18,125 CMUs genome-wide, occurring on all chromosomes and spanning a median of ~1 kb. Notably, 50% of CMUs had evidence of long-range correlation with other proximal CMUs. Although the size and number of CMUs varied across datasets, we observed strong intra-tissue consistency among CMUs, with those in testis encompassing those seen in most other tissues. Approximately 20% of CMUs were highly conserved across normal tissues (i.e. tissue independent), with 73 loci demonstrating strong correlation with non-adjacent CMUs on the same chromosome. These loci were enriched for CTCF and transcription factor binding sites, always found within putative TADs, and associated with the B compartment of chromosome folding. Finally, we observed significantly different, but highly consistent, patterns of CMU correlation between diseased and non-diseased states. Our first-generation, genome-wide, DNA methylation map suggests a highly coordinated CMU regulatory network that is sensitive to disruptions in its architecture.

## Introduction

Methylation of DNA CpG dinucleotides is an important regulator of gene transcription(Attwood, Yung et al. 2002), and through intermediary biochemical and physiologic processes, a crucial transducer of environmental stimuli including, nutrition (Anderson, Sant et al. 2012) and toxins (Szyf 2011). More recent studies have reported associations between quantitative variation in bulk tissue-level methylation and a variety of diseases and traits (Wei, Tao et al.). This differential methylation between affected and unaffected individuals is thought to result in changes in gene expression that relate to the underlying pathophysiology. At the same time, DNA methylation shows extensive variation between different tissues and different cells of the same tissue, making interpretation of these associations challenging.

An added level of complexity is that DNA methylation at adjacent CpG sites is known to be highly correlated (Liu, Li et al. 2014), suggesting that such methylation is also systematically organized and coordinated at the inter-CpG level. At the genome scale this organization is reminiscent of the highly coordinated folding of DNA at the epigenomic level, and of ‘blocks’ of correlated single nucleotide polymorphisms (SNPs) in strong linkage disequilibrium. However, specific examples of inter-CpG correlation are few (Gunasekara, Scott et al. 2019), meaning that the extent, pattern, interindividual and inter-tissue variation, and disease-specificity of methylation correlation remains unknown. Understanding the consistency of these patterns across individuals and tissues and placing them in the context of other genome features are a prerequisite to deeper explorations of the epigenome and its relationship to gene regulation.

Defining methylation patterns at genome-scale resolution has several inherent challenges – variation at the tissue (and cellular) level, definitions of correlation boundaries, and temporal variation with age are all uncertain, but potential, confounders. At the same time, differences in inter-CpG correlation between disease states might indicate qualitative differences in methylation coordination that could impact regulation and be relevant to phenotypic variation (Hoffman, Bendl et al.). Here, we leverage advances in image processing to implement a novel image clustering method for exploring inter-CpG methylation correlation, which we term correlated methylation units (CMUs). We apply our method to genome-wide DNA methylation data from the Illumina 450K/EPIC bead chip in 12,744 individuals from publicly available- and in-house datasets assayed across 26 different human tissues. We map proximal (-*cis*) and distal (-inter-*cis*) CMU variation across tissues, annotate tissue-independent CMUs with 2D and 3D regulatory maps, and determine qualitative differences in CMUs between disease and control states.

## Methods

### Samples and Datasets

We utilized 35 publicly available DNA methylation datasets profiled using either Illumina’s HumanBeadchip 450k or EPIC beadarray. In order to have sufficient interindividual variation to determine and characterize correlation, included studies needed to include at least 30 subjects. The final study datasets included 32 cancer-based studies from The Cancer Genome Atlas and two blood-based studies (Lehne, Drong et al. 2015)(Konigsberg, Barnes et al. 2021), alongside our in-house buccal (epithelial)-based study of severe acute malnutrition (SAM) (Schulze, Swaminathan et al. 2019) (Table 1, **Supp. Table 1**). The majority of these cohorts were sampled across multiple independent tissues, and included participants ranging in age from infancy to 80 years old with roughly equal gender distributions within a study, with the exception of gender specific studies (e.g. BRCA, PRAD; **Supp. Fig. S17**). Out of 32 TCGA 450K Illumina cancer studies 11 also contained data (for more than 30 individuals) from tissue ostensibly unaffected by cancer, which we refer to as ‘normal tissue’.

### DNA Methylation

All cases were surveyed using either the Illumina 450K array, or its updated incarnation, the Illumina EPIC array. Details of these platforms are well described, but in summary, they consist of ~450,000 (450K array) or 800,000 (EPIC array) oligonucleotide probes at single-CpG resolution spaced across the genome to cover known promoters, enhancer, and putative regulatory sites (Bibikova, Barnes et al. 2011), initially generated to cover promoter regions derived from cancer-based studies (450K), and subsequently updated to include additional enhancer sites from subsequent studies (EPIC). CpG sites assayed on the 450K array are largely a subset of the EPIC array, with >90% overlap (Solomon, MacIsaac et al.).

### Data Normalization and Filtering

Our analysis pipeline was designed to work from pre-processed beta values with, at a minimum, standard quality control of samples and probes (e.g. removal based on detection p values and data failures), background correction, type 1 and type 2 control of color bias, and confounder adjustments for batch, plate, chip, etc. as outlined in (Wilhelm-Benartzi, Koestler et al. 2013). For the datasets used here, we proceeded from the primary data processing and normalization as described in the accompanying manuscripts (TCGA, Blood - (Lehne, Drong et al. 2015), (Konigsberg, Barnes et al. 2021) and SAM (Schulze, Swaminathan et al. 2019). In order to mitigate against the undue influence of outliers in the datasets, we converted probe-wise sample distributions to normal distributions using qqnorm in R. The analysis pipeline is also designed to utilize post-hoc filtered CpG sites from raw data. In this proof of principle study, we limited our analyses to autosomal regions to obviate the added complexity of methylation patterns on the sex chromosomes.

### Image Clustering Method (ICM): Identifying Correlated Methylation Units (CMUs)

**Figure 1.**
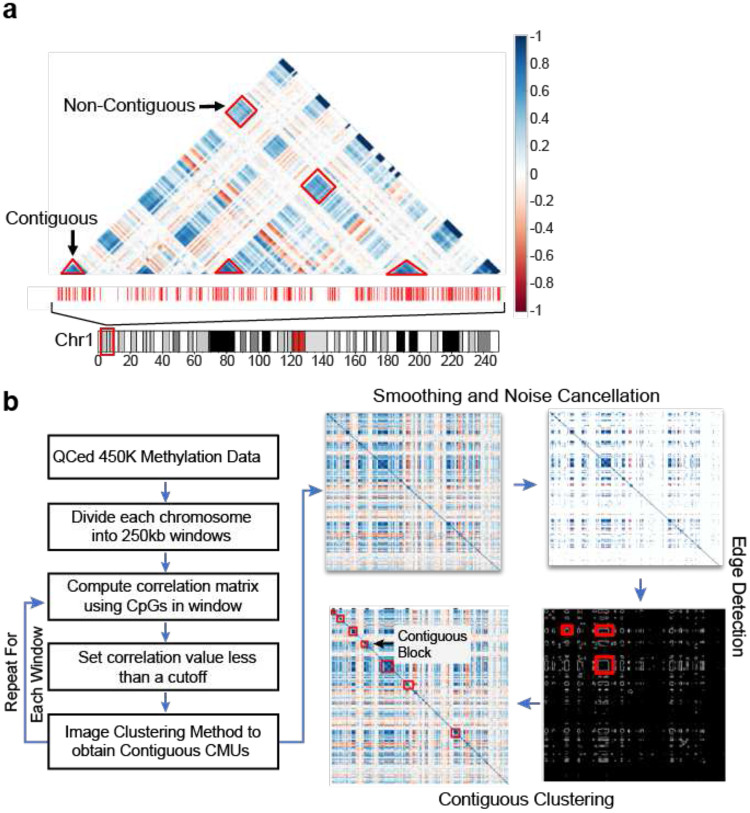
CMU (Correlated Methylation Unit) and Image Clustering Method (ICM) **1a**. An example of a correlation matrix in a 250kb genomic region, with CpGs arranged in genomic order. Red thin bars show genomic distribution of 450k CpG probes in that window. The colors in the triangle heatmap represent pearson correlations for CpG pairs(see methods). Densely colored regions outlined with red edges forming triangles and rectangles are examples of observing contiguous and non-contiguous CMUs, respectively. **1b**. Illustration of ICM steps. A correlation matrix is smoothed and then a threshold is applied on it. The image gradients are calculated and boundaries in the gradient are used to identify CMUs. Correlated CMUs are grouped together to form non-contiguous CMUs (see methods and Supp Fig S2).

We divided quality-controlled methylation data into non-overlapping windows, arbitrarily set to a genomic distance of 250 Kb. Pearson correlations of methylation profiles of all possible pairs of probes within each window were then calculated (Fig. 1), resulting in a correlation matrix for each window. The size of a correlation matrix (symmetric and square) for a window depends on the number of CpG probes encompassed, which varied from window to window. Each 250kb-window correlation matrix was treated as an image, with each pixel representing the strength of correlation bounded by the extremes of negative (−1) and positive (+1) correlation. The image was then smoothed using box filtering with unequal weights (Fig. 1, **Supp. Fig. 2**). To reduce the noise in the system, values below a minimal cutoff were set to 0. When working with a given (individual) cohort we found a correlation cutoff of 0.6 to provide a sufficient balance between noise and signal; however, this cutoff can be adjusted depending on sample size and other factors such as knowledge and existence of heterogeneity of cell types of the tissue of interest, or the goal of the study – e.g. higher cutoffs can be used to identify more tightly correlated methylation units that could be interpreted as sub-CMUs (**Supp. Fig.3**). For example, we utilized 0.4 cutoff for studying CMUs across tissues in order to provide larger regions that could be evaluated across tissues.

After setting a cutoff threshold, we then computed image gradients to identify edges in the image. Rectangular edges then lead to the identification of contiguous (genome proximal) and non-contiguous CMUs (interspersed with non-correlated sites). Two or more contiguous CMUs with strong positive or negative correlation with each other but interspersed by CpG sites without strong methylation correlation (unsynced units) are then referred to as non-contiguous CMUs (Fig. 1). In this classification, our underlying assumption is that CpG sites within a CMU that are not assayed by the array (Illumina 450k or EPIC array) are also in sync with the interrogated CpG sites. In preliminary studies, we found this to generally hold true. Deviations from this assumption should only affect the size classification of the underlying CMUs (i.e. create smaller units), but should not substantively impact the functional interpretations of CMU presented in this paper.

### Tissue Independent contiguous and non-contiguous CMUs

Each TCGA normal group was independently processed using ICM to obtain group-specific contiguous and non-contiguous CMUs. Genomic regions constituting contiguous CMUs in more than 80% of unique tissues (when more than one dataset is available for a tissue, we chose the one with largest sample size) were identified as conserved contiguous CMU genomic regions (**Supp. Fig. 4**). For the purpose of studying conserved non-contiguous CMUs across tissues we limited our search to non-contiguous CMUs that consisted of 3 or more contiguous CMUs and defined the genomic region under consideration as occurring between the first bp position of the first CMU (in genomic linear order) and end bp position of the last CMU. As above, genomic regions constituting non-contiguous CMUs in more than 80% of the tissues were identified as conserved non-contiguous CMU genome regions (Supplementary Figure 4). Bedtools (Quinlan and Hall 2010) was used to identify intersecting regions for both conserved contiguous and conserved non-contiguous CMUs.

### Asymmetric Similarity Measure between 2 sets of CMUs

To compare genomic regions of two ICM defined sets of CMUs, say $S_{1}$ and $S_{2}$ (arising from different cohorts, tissues, parameters, etc. ) we define an asymmetric similarity score that can also be viewed as a Tversky index (Tversky 1977) with parameters α=1 and β=0.


$$A_{sy}(S_{1},S_{2})=\frac{\#\;of\;CMU\;in\;S_{1}\;that\;intersect\;(genomic\;region)\;with\;some\;CMU\;in\;S_{2}}{\#\;of\;CU\;in\;S_{1}}$$

### Annotation of Regulatory Features

We utilized the Ensemble regulatory feature database, derived from the *Regulatory Build* process as described in (Zerbino, Wilder et al. 2015). Overlap between a regulatory feature region and a CMU region was annotated using *bedtools*. Ensemble was used to determine regulatory features: Promoter, Promoter Flanking, Enhancer, CTCF, TF and open Chromatin. CpG sites on the 450K Illumina are biased in their placement (Bibikova, Barnes et al. 2011), which could bias feature enrichment analysis; therefore, we generated random sets of CMUs (background control CMUs) to mirror the observed/actual CMUs. To determine whether specific regulatory features were enriched in a defined CMU, we used two tests: a CpG *probe-based* test and a *region-based* test (**Supp. Fig. S13**). For region-based tests we used a comparison of ‘background’ random regions chosen without regard to their inter-CpG correlation. These were identified by first obtaining the number of CMUs found by the ICM algorithm, then removing windows with insufficient CpG sites (<4 [lenient] or <10 [stringent] sites) in the ICM analysis. We then chose n windows randomly out of the remaining (450K-contained) windows. Within each of these random windows, we then picked a region matching the genomic size of the CMU under test to act as a background control for each observed CMU. These random background CMU units were annotated as described above. The number of times a feature was annotated by this test CMU was then made relative to annotations of the random background CMU units. As an example, if “CTCF region” was annotated five times, it receives a score of five (5). The background/control group of CMUs matching the observed CMU is then generated 10,000 times, producing a distribution of scores for each feature. These sampling distributions were then modeled as normal distributions to compute a likelihood of observing the actual score. For sets of CMUs within individual cohorts, we also performed hypergeometric tests comparing the number of probes collectively across CMUs annotated for a specific regulatory feature with the number of 450K probes annotated for that regulatory feature. For CpG probe-based tests, we simply use a Fisher test on contingency tables of CpG probes in a feature for CMUs and Illumina CpG site set (**Supp Fig. S13**). Region-based tests cannot always precisely match regions for the density of CpGs and so may produce inflated results. CpG probe-based tests, on the other hand, cannot account for spatial proximity of CpG probes and can produce inflated results for CpG island, promoter and related regions; therefore, the two tests provide complementary data.

### Annotation for Spatial organization of Chromatin

Topologically Associated Domains (TADs) and chromatin loop annotations across different tissues were downloaded from 3D genome browser (Wang, Song et al. 2018), which uses the methods of (Dixon, Selvaraj et al. 2012) for TAD calling and of (Salameh, Wang et al. 2020) for loop calling. For replication purposes we also used TAD annotations from (Liu, Porter et al. 2019), which, along with Dixon et. al., includes a TAD calling method that uses GMAP (Yu, He et al. 2017) and IS (Chen and Lei 2019) TAD calling methods. A/B compartment and FIREs (Frequently Interacting Regions) data was downloaded from (Schmitt, Hu et al. 2016). To understand the overlap between CMUs and chromatin features, we wanted to also contextualize the overlap between Illumina CpG sites and chromatin features; therefore, for a given set of CMUs and a chromatin feature we calculate the following parameters:

$$\%\;of\;CpG\;overlap=\frac{\#\;of\;Illumina\;CpG\;sites\;overlapping\;a\;chromatin\;feature\;region}{total\;\#\;of\;Illumina\;CpG\;sites}$$

$$\%\;\text{of CMU overlap}=\frac{\#\;of\;CMUs\;that\;overlap\;one\;or\;more\;chromatin\;feature\;regions}{total\;\#\;of\;CMUs}$$

$$\%\;\text{of unique}\;overlap=\frac{\#\;of\;CMUs\;that\;overlap\;only\;one\;chromatin\;feature\;regions}{\#\;of\;CMUs\;that\;overlap\;one\;or\;more\;chromatin\;feature\;regions}$$

### Tissue specific CMU overlap comparison for A/B compartment

We computed the above values for each pair of tissue specific non-cont CMUs and tissue specific A/B compartments and combine these to provide a tissue pair score:

$${\text{pair(CMU}}_{\text{tissue}},A_{\text{tissue}})=\log\;(\frac{\%\;CMU\;overlap\;with\;A\_tissue}{\%\;CpG\;overlap\;with\;A\_tissue})$$

The same computation was repeated for annotations of the B compartment. If CMUs are found in A and B compartments with equal frequency, then this score will be similar across both compartments.

### Differential CMUs

To determine the potential for differential CMU between any two comparison groups, we first separated each cohort into cases and controls. This approach requires that either the samples are collected from a homogeneous tissue and/or that tissue/cell proportions are known or can be robustly estimated. Normalized methylation data (Y) was regressed on confounding factors (X) (e.g. age, gender, cell/tissue proportion for non-homogeneous data, etc.); Y=aX+R. The residuals (R) of this regression were then used to compute pairwise correlations, also known as partial correlations. ICM was then used for each group separately to identify CMUs as described above. For each CMU defined within a given group, a corresponding paired correlation matrix is compiled in the other group using the same CpGs. In this way each group-defined CMU is represented in cases and controls. The correlation matrices within each pair are then compared to identify CMUs that are significantly more correlated in the CMU- defining group when compared to the other group.

A CMU is identified as showing significant differential correlation if it passes multiple correction p-values for two tests - the first is a correlation matrix comparison test derived using the cortest function in R (Revelle 2020) with inputs: R1 as CMU correlation matrix for the CMU defining group, R2 as the corresponding matrix for the other group, n1 number of samples used to compute R1, n2 number of samples used to compute R2 and Fisher=TRUE. This test is based on a chi square comparison proposed by Steiger to study correlation matrices (Steiger 1980). Chi-square statistics are computed on Fisher transformed correlation values, accounting for sample sizes of both groups and using a degree of freedom that depends on the number of CpG probes present in the CMU. The null hypothesis was that the off-diagonal entries of the difference between the two correlation matrices (R1-R2) would be zero. The p-values are corrected using Bonferroni correction for each dataset separately. The second test compares off-diagonal entries using Wilcox rank sum test (implemented in R as wilcox.test function providing input paired=FALSE). The alternative hypothesis is that the distribution of the diagonal entries of the CMU defining group is shifted to the right of its counterpart by mu or more (correlation level difference); for the sample sizes used here, we chose mu= 0.1 as an arbitrary but sensitive cut-off. An indirect way of proxying for sample sizes of the groups can be done by varying mu. P-values are again corrected using Bonferroni correction. To be defined as significantly differentially correlated, both tests must be passed. Here we report effect size and p-values of Wilcox test for ease of explanation. To include non-contiguous CMUs in the analyses, we collapsed a set of CMUs and a set of non-contiguous CMUs into one list by removing the CMUs that contribute to non-contiguous CMUs.

## Results

### Image Clustering captures Correlated Methylation Units (CMUs) on the 450K/EPIC array

In order to perform a genome-wide scan for Correlated Methylation Units (CMUs), we first divided the genome into non-overlapping windows spanning 250kb. Strongly correlated CpG sites have been estimated to extend 10kb on average (Liu, Li et al. 2014); therefore, we chose 250kb to allow us to capture multiple potential CMUs in a computationally tractable manner. For the Illumina 450K array there are ~7000 windows across the autosomal genome (**Supp. Fig. S1a**) with the number of CpG sites within a window varying from a median of 37 to a maximum of 1300. The highest CpG probe density was observed at the HLA region of chromosome 6, with higher CpG densities around chromosome telomeres (**Supp. Fig. S1b**). To determine CMUs, we calculated Pearson correlation between all pairs of probes within the 250kb window, resulting in a correlation matrix for each window (Fig. 1a); a total of ~22 million CpG pairs across all windows were assessed.

Instead of binning CpG sites based on correlation between immediate adjacent sites and imposing a hard correlation score threshold, we used an Image Clustering Method (ICM) (Methods) to identify CMUs based on the presence of an overall strong correlation amongst CpG methylation profiles within a CMU even if the correlation was not high between every CpG pair within the CMU. This follows from ICM treating the correlation matrix in each 250kb-window as an image, with each pixel representing a correlation score between −1 and +1 (Fig. 1b). The image was then smoothed using pseudo box filtering (**Supp. Fig. S2**). The values of image pixels below a threshold a were set to 0. Image gradients were then computed to identify edges where the correlation between methylation profiles of adjacent CpG sites fell to zero, and to capture contiguous CMUs (at least 4 adjacent CpGs with strongly correlated methylation profiles), and non-contiguous CMUs where there was significant correlation between methylation profiles of non-adjacent CMUs (Fig. 1b). Imposing a particularly “low” (<0.4) correlation threshold a reduces the noise in the system; higher correlation thresholds result in smaller (or sub-) CMUs that are part of the larger CMUs observed at lower thresholds (**Supp. Fig. S3**). In our model, we chose 0.6 as the ‘default’ correlation to balance between these two extremes; consistent with this, we observed non-contiguous CMUs defined using a higher threshold often fell completely within a lower threshold-associated contiguous CMU (**Supp. Fig.S3**).

**Figure 2.**
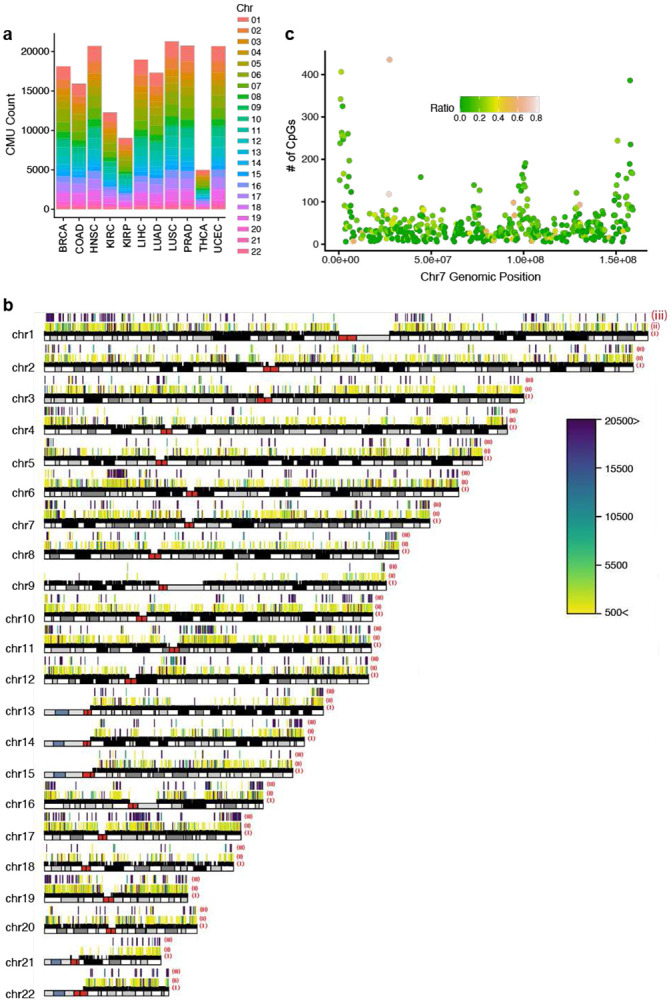
CMU Characterization **2a.** Distribution of total number of CMUs across TCGA studies for normal (non-tumor) samples. Each vertical bar corresponds to a study and the stacked colors represent chromosomal contribution to the total CMU count. **2b.** Ideogram showing genomic distribution of CMUs for KIRP (kidney renal papillary cell carcinoma) normal (non-tumor) samples. Three rows above each chromosome represent i. - location of 450k probes (closest to ideogram in Black), ii. location of contiguous CMUs (middle row), iii. location of non-contiguous CMUs (farthest row). The colors represent CMU sizes in base pairs (bps). **2c.** Distribution of number (#) of CpGs identified in any CMU in a 250kb window across chromosome 7 for KIRP normal (non-tumor) samples. Each dot represents a 250kb window; x axis shows the genomic position of the window and y axis represents number of associated CpGs in that window. Colored dots show proportion of all 450k probes contributing to any CMU (Supp Fig S7;S4 & S8; S5 & S6)

We used ICM to identify CMUs across 35 in-house and publicly available cohorts (Table 1, **Supp Table 1**). Starting with eleven (11) groups comprised of normal (non-tumor) samples from TCGA as our baseline discovery set, ICM defined a median of 18,125 contiguous, dataset-specific CMUs (range: 4993-21,279), with a median length of 860 basepairs (bps) (IQR: 381bp to 3,423bp) (Fig. 2A). These CMUs were found to be broadly distributed across the genome in each tissue (Fig. 2B, **Supp. Fig. S4**), with the HLA region on chromosome 6 generally having the most CMUs (**Supp. Fig. S5**). On average, ~30% of all CpGs were contained within one of the CMUs identified (**Supp. Fig. S6**), and each CMU consisted of a median of 6 CpG sites (range 5-300; Fig. 2C). The overall distributions for the number of CMUs, the number of CpGs within a CMU, and CMU length in cancer tissues were comparable with their respective normal tissues (**Supp. Fig. S7**).

We noted that about half of the CMUs within a given dataset had evidence of correlation with near-by CMUs. We formally denoted these as non-contiguous CMUs (**Supp. Table 3**), where two adjacent CMUs are interspersed with CpGs that belong to neither CMU. In total, there were a median of 3,689 non-contiguous, dataset-specific CMUs (range: 586-4,789), with each non-contiguous CMU having an average of 3 CMUs per 250kb window (**Supp. Table 4**). As expected from the distribution of CMUs, non-contiguous CMUs were also broadly distributed across the genome in each tissue (Fig. 2B, **Supp. Fig. S9**).

### CMUs are consistent within a given tissue and have variable cross-tissue overlap

**Figure 3.**
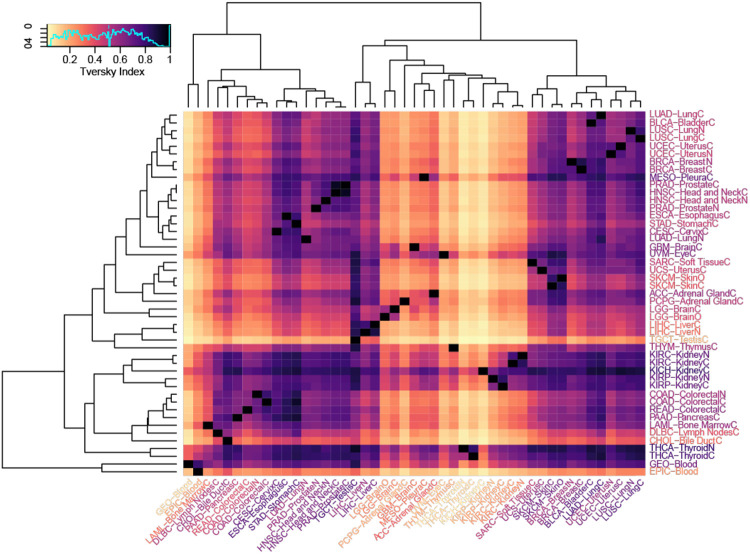
CMU representation within and across tissues Heatmap of asymmetric square matrix of Tversky scores (Asy(S1, S2); see methods, Supp Fig S10) computed for each possible ordered pair of datasets in Table 1. Horizontally placed tissue labels on the bottom (x-axis) corresponds to the first dataset in the ordered pair (S_1_) and vertical tissue labels on the right (y-axis) corresponds to the second dataset in the ordered pair (S_2_). Both sides of labels are colored with median column and row scores respectively and the dendrograms are based on hierarchical clustering (distance=1-cor and complete-linkage).

To understand the consistency and interrelationship of CMUs across tissues, we introduced an asymmetric similarity measure between any given ordered pair of CMUs S_1_ and S_2_: A_sy_(S_1_, S_2_), which varies between 0 and 1 (methods, **Supp. Fig. 10**). We applied this to every possible ordered pair of available tissue datasets (Table 1), from which we derived an asymmetric square matrix that quantifies the representation of CMUs both across and between tissues (Fig. 3, **Supp. Table 5**). Varying the second tissue for a fixed first tissue S_1_, A_sy_(S_1_, *), quantifies the representation of the first (S_1_) tissue’s CMUs in those of the other tissue, whereas A_sy_(*,S_1_) quantifies how representative the second tissue’s (S_1_) CMUs are of CMUs found in the other tissues. We see a variation in these scores that ranges between 0.16 to 0.8 (median A_sy_(S_1_, *)) and .08 to .76 (median A_sy_(*,S_1_)), which indicates presence of different degrees of unshared (tissue specific) CMUs and shared CMUs among tissues.

CMUs identified in kidney, pleura, thyroid had the highest presence in other tissues (median A_sy_(Kidney,*) between 0.7 and 0.8, median A_sy_(Thyroid,*)=0.75, median A_sy_(Pleura, *)=0.75). CMUs of kidney, blood and thyroid were least representative of other tissues (median A_sy_ (*,blood)=0.09, A_sy_ (*,Kidney)=0.08 and A_sy_ (*,Thyroid)=0.08 ) (Fig 3). Testis stood out among all tissues as being the most representative of CMUs found across our datasets (median A_sy_(*,Testis)=0.76). This is consistent with the germline haploid nature of most testicular tissue. Further, hierarchical clustering, based on CMU asymmetric similarity measures, clustered pairs of the same tissue, even from different studies (e.g. studies of kidney tissue: KICH,KIRC and KIRP, studies of colorectal tissue: COAD and READ, studies of peripheral blood: 450k chip and EPIC chip), as well as most biologically paired tissues (normal and cancer) within in a study (Fig 3), giving additional weight to our methodology.

### CMU conservation across tissues is commonly observed

**Figure 4.**
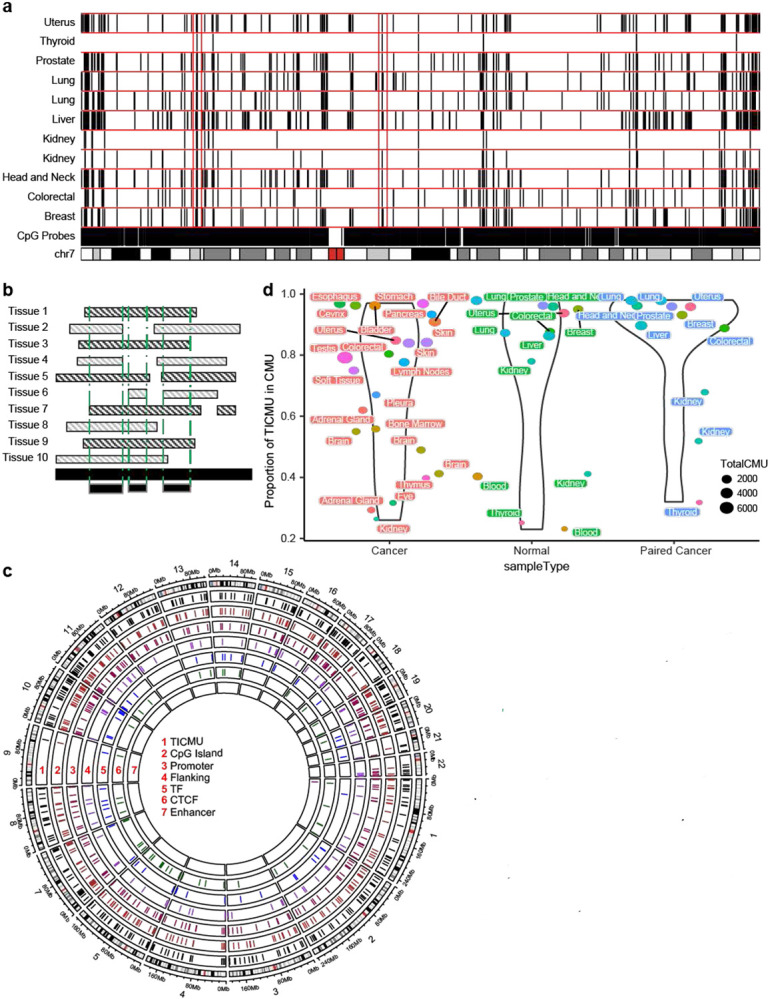
Tissue-Independent CMUs **4a.** CMUs along chromosome 7 for normal TCGA tissue samples with rectangular red boxes illustrating regions for which ICM identifies CMUs across the majority of tissues (>80%, methods). **4b.** Identification of tissue independent CMUs. The unbroken horizontal black bar at the base represents CpGs across a given genomic region. Alternate grey and black hatched bars represent genomic regions of CMUs of tissues in consideration. Bottom most black bars outlined in grey represent the genomic regions identified as TI CMUs. **4c.** Circos plot of TI CMU regions in the outer most panel (black). TI CMU annotation for regulatory regions is displayed in the corresponding regulatory circular inner panels. **4d.** TI CMU representation in tissue CMUs using Tversky index (y axis; methods). Each dot corresponds to a dataset, split in 3 categories (x- axis) unpaired cancer tissues, normal tissues, and paired cancer-normal tissues (see Table 1).

The high asymmetric scores seen between tissues suggested that the information content and utility of CMUs may be partially independent of the tissue; therefore, to provide a context for interpretation across tissues, we looked for CMUs that were present across most tissues. For this evalation, we excluded the highly-complex HLA region, given the extensive polymorphism and uncertain methylation readouts from array-based platforms in this region (Rakyan, Hildmann T Fau - Novik et al.). Further, we focused on higher confidence CMUs by restricting our analysis to CMUs with >10 adjacent correlated CpG sites (as opposed to the more liberal >4 adjacent CpG sites used in our initial analyses) (**Supp. Fig.8**) and looked for the largest region of overlap across each tissue-defined CMU (Fig. 4A). We defined tissue-independent CMUs (TI-CMUs) as CMUs observed in more than 80% of unique normal TCGA tissues (Fig. 4B). We found 473 TI-CMUs across the genome (Fig. 4C, **Supp. Table 6**), representing ~20% of dataset-specific CMUs. Consistent with the asymmetric similarity score, thyroid, kidney, and blood showed the most CMU overlap with TI CMUs (up to 50%; **Supp Fig.11**), and TI CMUs identified using only normal tissues had a comparable overlap with CMUs of both their paired cancerous tissue, and of other cancer tissues (Fig 4D).

**Figure 5.**
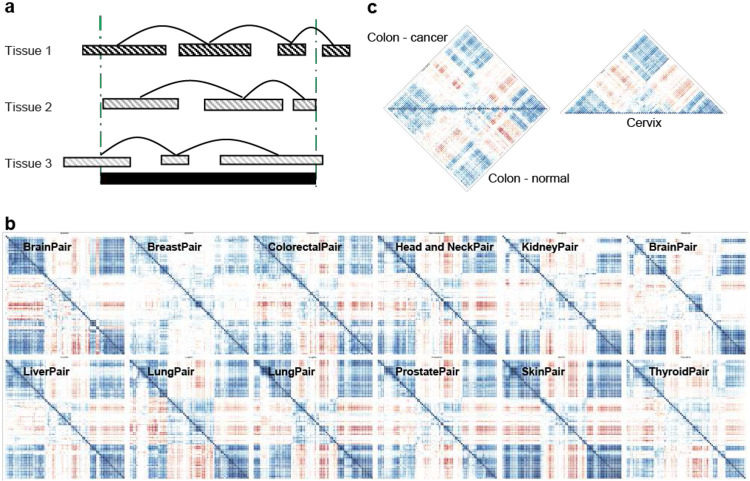
Non-contiguous Tissue Independent CMUs **5a.** Illustration of identification of tissue independent non-contiguous CMUs. The shaded bars represent contiguous CMUs with correlated CMUs connected by lines (i.e. non-contiguous CMUs). Dotted green lines identified a TI non-contiguous region (see methods). **5b.** Example of a non-contiguous TI CMU region on chromosome 5 (chr 5q15: 93570534- 93596339, hg38), which shows three correlated units with the middle one showing negative correlation with the other two across multiple tissues. The first 3 rows are cancer tissues mirrored along diagonal; lower two rows display studies with both normal (above diagonal) and cancer (below diagonal) tissues. **5c.** Detailed depiction of colon and cervix correlation matrix for the same region shown in Fig 5B

Next, we sought to investigate tissue-independent long-range correlation of methylation with an expectation that CMUs within a non-contiguous CMU may not have the same boundaries across tissues. Therefore, we identified tissue-independent (TI) non-contiguous CMUs by simple intersection of genomic regions (defined by the start of first CMU under consideration and end of last CMU under consideration) across tissues (Fig. 5A). This approach necessarily allows for variation in CMU boundaries within tissue-specific non-contiguous CMUs. Despite this, at a given genomic locus, several non-contiguous TI CMUs had a similar distribution of CMUs across many tissues (Fig. 5B, **Supp Fig. 12**). In total we obtained 74 TI non-contiguous CMUs (**Supp. Table 7**), with a region on chromosome 5q15 (93570534- 93596339, hg38), showing three consecutive CMUs wherein the middle one consistently negatively correlated with the other 2 adjacent CMUs within every tissue studied (Fig. 5C).

### TI CMUs are enriched for regulatory annotations and appear to link regulatory units

**Figure 6.**
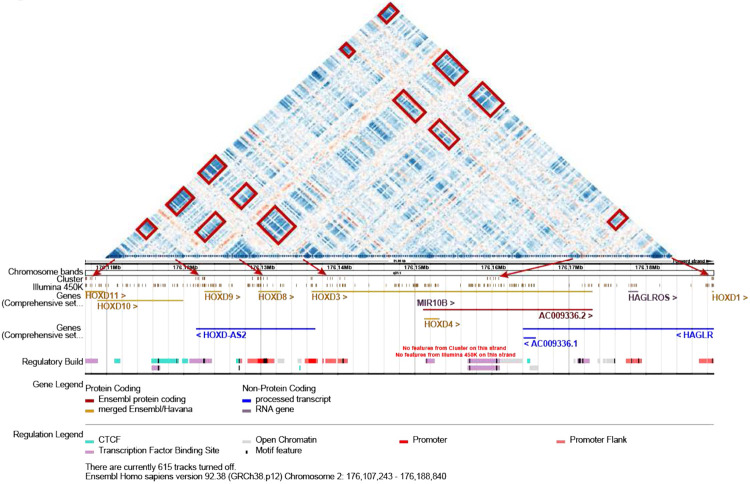
Example of a non-contiguous CMU linking regulatory units Correlation matrix for Hox gene cluster region (chr2: 176107243-176188840, tissue: GEO-450k in Table 1). Below the correlation map is an Ensembl browser track of 450k CpG probes (Illumina 450K) and non-contiguous CMU forming CpG probes (Cluster) for the same region. Red arrows identify correlated units and their corresponding genomic regions, alongside annotations of genes and regulatory build; this non-contiguous CMU overlaps multiple regulatory annotations.

**Figure 7:**
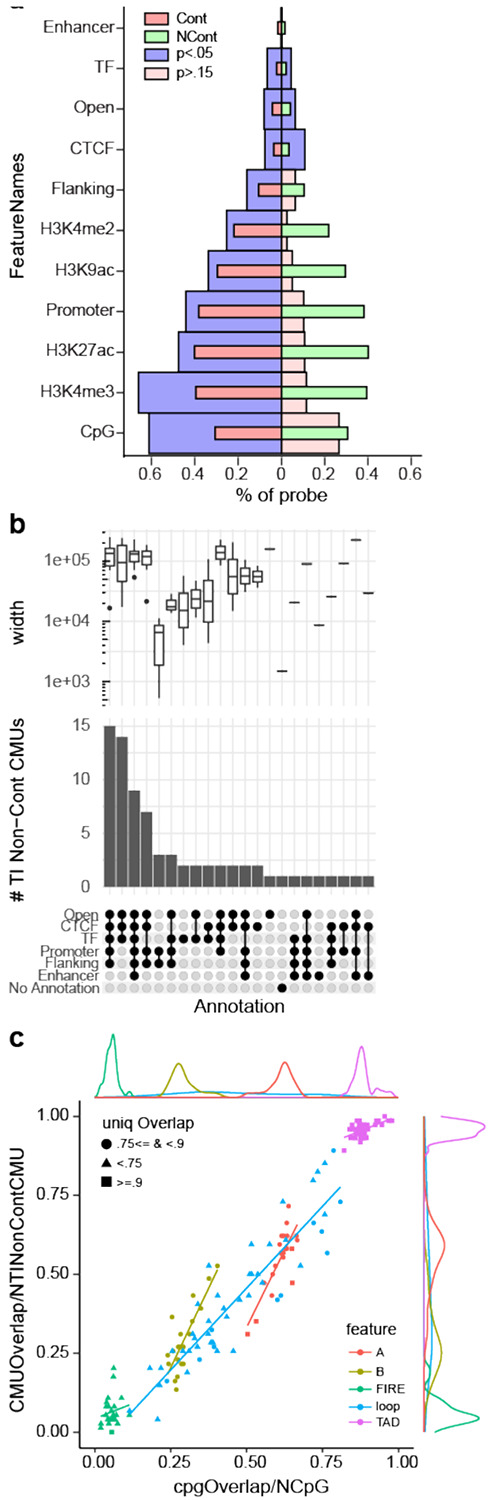
Enrichment of regulatory elements and chromatin structure among Non-contiguous TI CMU **7a.** Pyramid plot showing regulatory feature enrichment analysis using probe-based test (methods, Supp. Fig. S14). Left side corresponds to TI CMUs and right side corresponds to non-contiguous TI CMUs. The X axis shows percent overlap of CpG probes with a regulatory feature. Red and green bars represent proportion of 450k probes that overlap a regulatory feature - left and right side are mirrored. Purple and pink bars are the proportions of CpGs involved in CMU and non-contiguous CMU (respectively) overlapping a given regulatory feature, **7b.** Upset plot shows the observed combinations of regulatory units linked in non-contiguous TI CMUs. Bar plots depict number of non-contiguous TI CMUs that overlap the regulatory features marked below it (filled circle). The top panel shows size variation among corresponding CMUs (also see Supp Fig S14). **7c.** Proportion of non-contiguous TI CMUs that overlap a given chromatin feature (y axis) vs fraction of CpG probes that overlap with the chromatin feature (x axis) serving as a baseline (see methods). Each color corresponds to a different feature; with each feature dot representing an annotation for a unique tissue. Non-contiguous TI CMU can potentially overlap more than 1 annotation genomic interval of a feature, dot shapes represent different fraction levels of unique overlaps (i.e. proportion of overlap with only 1 annotation).

In annotating CMUs across the genome, we identified a non-contiguous CMU across the HOX gene family on chromosome 17 that appeared to link methylation overlying promoter regions and enhancers (Fig. 6); subsequently, we also noticed that many non-contiguous TI CMUs overlapped clusters of related genes (e.g. *PCDHA, PCDHB, SNORD, KRTAP,OR(Olfactory receptor family)*) (**Supp. Table 7**). This led us to hypothesize a potential link between regulatory units and CMUs. We utilized Ensembl [14] to annotate regulatory features of the genomic regions corresponding to TI CMUs (Methods). Relative to background Illumina CpG sites, we found TI-CMUs to be enriched across all Ensembl regulatory features except for enhancer regions (Fig. 7A). Specially, enrichments of CpG island and H3K4me3 were most prominent (probe-based test; see methods), and this was also found using a secondary method of assessing enrichment (**Supp. Fig.13**). Notably, only a small number of CpG probes are found over annotated enhancer regions on the 450K/EPIC arrays, and almost none of the resulting CMUs overlapped this feature. We note, however, that Ensembl ‘enhancer’ annotations overlap with promoter flanking regions and are collapsed into this latter category when the two overlap; thus, enhancer regions may not completely be devoid of CMUs.

By contrast, TI *non-contiguous* CMUs were enriched for transcription factor (TF) and CTCF binding sites, with the caveat that TF binding site annotations are the default for regions not included in any other primary annotations (i.e., promoter, promoter flanking, CTCF, and enhancer), and so are likely to include distant binding sites. CTCF binding sites are often linked to chromatin looping, suggesting a potential link between 3-dimentional spatial DNA folding and TI non-contiguous CMUs. We also observed that in contrast to TI contiguous CMUs, TI non-contiguous CMUs tended to overlap multiple regulatory regions (Fig. 7B). In part this is due to the larger genomic regions of TI-non-contiguous CMUs and the non-linear probe placement of the array, but alongside examples of positive and negative correlation between distant CMUs, this might also suggest long-range coordination between regulatory motifs.

### TI non-contiguous CMUs occur within TADs and appear more frequently in the B compartment

The long-range interactions of contiguous CMUs were reminiscent of regulatory Topographical Associated Domains (TADs) and loop domains described from Hi-C and related 4D genome folding assays. We thus sought to determine the relationship between TADs and our TI non-contiguous CMUs. When we looked at tissue specific TADs (from (Wang, Song et al. 2018), Methods) we found that even though the resolution of conserved TADs (~1Mb) is substantively larger than our correlation windows, almost all of our TI non-contiguous CMUs (>90%) were contained within TADs (i.e. did not cross an annotated TAD boundary) (Fig. 7C). For chromatin loops on the other hand, among the TI non-contiguous CMUs overlapping this annotated feature, more than 50% overlapped two chromatin loops (Fig. 7C), and this was true for almost all of the tissues assessed. This suggests a physical proximity of the two ends of the two overlapping chromatin loops (**Supp. Fig. 14**). Further, as expected for any annotation, there is a linear relationship between the fraction of TI non-contiguous CMUs overlaps and fraction of Illumina CpG overlap (Fig. 7C). But TADs have the smallest slope implying that even with less CpG overlap there is higher CMU overlap. We also found that for a given fraction of TI non-contiguous CMUs overlapping the A compartment, the fraction of CpG probe overlap with B compartment was much lower (Fig. 7C). To further investigate, we compared tissue specific CMUs overlapping A/B compartment and observed a similar pattern (**Supp. Fig.15**), implying that CMUs occur more frequently in the B than the A compartment.

### Differential correlation patterns between disease states highlight putative candidate loci

Finally, we explored the impact of disease-state on CMUs using buccal cell samples from children with severe acute malnutrition (SAM) (**Table 2**). We previously demonstrated that these childhood buccal samples are composed predominantly of buccal epithelial cells (median probability of predicted buccal samples: 0.84) (Schulze, Swaminathan et al. 2019); consequently, we anticipated that differences in CMU correlation patterns between childhood SAM cases (with edematous malnutrition) and controls (with non-edematous malnutrition) would be largely the result of phenotypic differences, rather than cellular composition. For this analysis, we also included covariates of age, gender and country.

**Figure 8:**
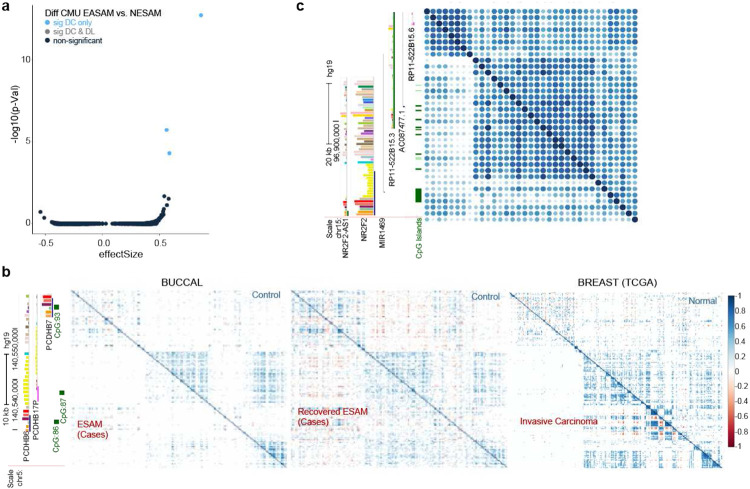
Differential correlation in SAM **8a.** Volcano plot of SAM differential CMUs identified for edematous- (cases) and non-edematous-(controls) malnutrition samples (see methods). Each dot represents a CMU, with blue dots having significant differentially correlated CMUs at a Bonferroni threshold for the number of tests. A negative effect size implies higher correlation in cases and a positive effect size the opposite (methods). **8b.** A differential CMU on chromosome 5 showing correlation patterns between non-edematous (above diagonal) and edematous (below diagonal) malnutrition subjects (left panel); non-edematous adults (above diagonal) and edematous adults (below diagonal) (middle panel) and normal (above diagonal) vs cancer (below diagonal) samples for BRCA study. **8c.** Example of a differential CMU where small CMU units in cases show correlation but lack of correlation among themselves. This was only observed in samples from Jamaica.

Three CMUs showed evidence of differential correlation – characterized in this first-pass analysis as weaker correlation between CpGs (Figure 8A & 8B, **Supp. Table 8**). This observation is consistent with wide-spread differential hypomethylation between Edematous SAM (ESAM) and Non-edematous SAM (NESAM) noted at specific CpG clusters in these samples (Schulze, Swaminathan et al. 2019); however, none of the differentially correlated regions showed statistically significant differential methylation. The strongest differential CMU signal was found on chromosome 5 overlying the *PCDHB* gene family (Fig. 8B). Interestingly, epigenetic changes in PCDH gene cluster families have been observed in studies of other traits, including prenatal alcohol exposure, child maltreatment, and psychiatric disorders, among others reviewed in (El Hajj, Dittrich et al. 2017). Unlike some of these studies of long-term epigenetic modification, we observe that buccal samples from adult survivors of SAM, who are temporally removed from the acute nutritional insult, showed the same pattern of correlation across this region in both cases (ESAM) and controls (NESAM), albeit with more ‘noise’ in the system, likely the result of the more mixed tissue composition in these samples (median probability of predicted buccal samples: 0.31). Further, this same CMU region in normal mammary tissue (TCGA) showed the same overall pattern of correlation as in buccal tissue, with evidence of differential correlation within breast cancer samples, which, in turn, had markedly a different pattern than observed in starved buccal epithelial cells, with newly correlated regions that included negatively correlated CMUs and non-contiguous correlation structures (Fig. 8B). This region also lies within a superTAD that is conserved between mouse and humans in neural tissue (Jiang, Loh et al. 2017). Despite evidence of environmental impact on these gene clusters and knowledge of chromatin state, a mechanistic understanding of epigenetic changes and their interplay with disease pathogenesis is still unknown. When considering Jamaican samples another interesting differential CMU was found over *NR2F2* also known as *COUP-TFII* (chr15: 96868857- 96935641). It has two small, strongly correlated CMUs in cases and controls that are correlated with each other in controls, but not in cases (Fig. 8C). *NR2F2* is a ligand inducible transcription factor involved in regulating multiple genes in many tissues (Polvani, Pepe et al.).

## Discussion

We provide a first-generation interrogation of genome-wide patterns of correlated DNA methylation across 26 unique tissues in ~12,500 individuals. We show that 30% of all CpGs on the 450K array belong to highly correlated methylation units (CMU). We find a wide range of variation in the genomic context of CMUs across tissues, with testis standing out as the biggest CMU superset. This gives some biological credibility to our observations – CMUs may be established in the germline and then further modified with tissue differentiation. We observed 473 tissue-independent CMUs (present in >80% of tissues), with evidence for long-distance correlation between CMUs that may have implications for coordinated regulation of transcription at genomic loci. We also provide evidence that CMUs can be disrupted in disease states, in a manner that is independent of differential methylation.

Previous overviews of methylation clustering have largely focused on either the quantitative aspects of the distance between pairs of methylation sites (Jaffe, Murakami P Fau - Lee et al.) or imposed an *a priori* context based on genomic location (Butcher and Beck). Our approach is agnostic to the underlying genomic features, even as we note an enrichment of promoter and transcription factor binding sites within our described CMUs. We posit our approach as ‘first-generation’, as we are cognizant that CMUs conserved across bulk tissues may or may not translate to CMUs at the cellular level, for which cell deconvolution (cell-type specific) or the broader application of methylation to single cell technologies is needed. The application of the scanning method we employ here will be highly useful for existing array-based datasets and for emerging datasets of whole-genome bisulfite sequencing. We also anticipate that the screening method itself has scope for improvement in subsequent iterations. For example, in the present state ICM captures CMUs on pre-set parameters but it does not learn from captured CMU patterns. As we improve our understanding of plausible CMU patterns by utilizing more and more datasets and expend our biological understanding, ICM can be further refined by employing deep learning methods (Alzubaidi, Zhang et al.).

A particular limitation of such genome-wide screens is that the biological implications are not inherently clear. We note that our CMUs are contained within established TADs, but the extent of the relationship between TADs and other loop domains to CMUs, the mechanisms involved in maintaining and establishing such correlation structures, and the regulatory sequelae of disrupting CMUs, are unclear. A similar conundrum is the impact of *cis*- and *trans*- genetic variation - methylation quantitative trait loci are major considerations for single site methylation, and presumably will have a similar role across CMUs, although this remains an empirical question for the field.

Despite the mechanistic black-box of CMUs, our study implies that they are not simply the result of statistical stochasticity. Rather, the structured view of methylation, particularly at CMUs suggests that, like common SNPs, there is redundancy between methylation sites that can be exploited in designing more streamlined methods of genome-wide assessments of DNA methylation. Opening doors for developing “CMU-pruning” methods, like LD-pruning, with applications to EWAS, imputing, and cell proportion estimates. We also see that differential correlation of CMU between cases and controls appears to be non-random and does not always overlap sites of inferred differential methylation. Given the large number of already generated differential methylation case-control studies that have utilized the 450K/EPIC array, it seems likely that differential correlation also holds unique biological insights that can be derived from already generated datasets.

## Supplementary Material

Supplement 1

## Figures and Tables

**Table 1.** Number of individuals and tissues used from public-datasets

	TCGA																																Others		
# of Subjects	ACC	BLCA	BRCA	CESC	CHOL	COAD	DLBC	ESCA	GBM	HNSC	KICH	KIRC	KIRP	LAML	LGG	LIHC	LUAD	LUSC	MESO	PAAD	PCPG	PRAD	READ	SARC	SKCM	STAD	TGCT	THCA	THYM	UCEC	UCS	UVM	GEO-450k	GEO-EPIC	# of Groups
Disese Tissue	80	418	791	307	36	313	48	185	140	528	66	324	275	140	516	377	473	370	87	184	179	502	98	261	104	395	150	507	124	438	57	80		164	33
Normal Tissue			96			38				50		160	45			50	32	42				50						56		46			2664	296	13
Tissue Name	Adrenal Gland	Bladder	Breast	Cervix	Bile Duct	Colorectal	Lymph Nodes	Esophagus	Brain	Head and Neck	Kidney	Kidney	Kidney	Bone Marrow	Brain	Liver	Lung	Lung	Pleura	Pancreas	Adrenal Gland	Prostate	Colorectal	Soft Tissue	Skin	Stomach	Testis	Thyroid	Thymus	Uterus	Uterus	Eye	blood	blood	
