# Supplementary material for: A first-generation genome-wide map of correlated DNA methylation demonstrates highly coordinated and tissue-independent clustering across regulatory regions
Source: Res Sq. 2023 May 15:rs.3.rs-2852818. Preprint. [Version 1] doi: 10.21203/rs.3.rs-2852818/v1 (PMC10275061; doi:10.21203/rs.3.rs-2852818/v1)
Supplement: Supplement 1 [file NIHPPrs2852818v1-supplement-1.pdf]

SUPPLEMENTARY MATERIALS:

Fig S1 Distribution of 450k Illumina probes on 250kb resolution

Fig S2 Pseudo Box Filtering of correlation matrix/image

Fig S3 Impact of correlation threshold on CMU boundary

Fig S4 Ideogram plots of Contiguous CMUs for each normal tissue

Fig S5 Tissue-wise comparison of total number of CpGs forming CMU per 1M genomic window

Fig S6 Tissue-wise comparison of fraction of 450k probes forming CMU per 1M window

Fig S7 CMU characterization across all the datasets including cancer

Fig S8 CMU (>=10 sites) characterization across all the datasets including cancer

Fig S9 Ideogram plots of Non-Contiguous CMUs for each normal tissue

Fig S10 Tversky Index calculation

Fig S11 Proportion of CMU in TICMU

Fig S12 Non-Contiguous TICMU correlation pattern across tissues

Fig S13 Enrichment Test Illustration

Fig S14 Pyramid plot for region-based enrichment test & upset plot for contiguous TICMU

Fig S15 Regulatory enrichment for tissue specific CMUs

Fig S16 CTCF enrichment & illustration of non-unique loop overlap

Fig S17 CMUs in active (A) and non-active (B) compartments

Fig S18 Demographics of TCGA datasets

Link to supp table (https://www.dropbox.com/s/g898nqgrvc4mann/Tables.zip?dl=0)

Supp Table 1 - Tissue Details for TCGA

Supp Table 2 - CMU For Tissues

Supp Table 3 - CMU Ratios Forming Non-Contiguous CMUs

Supp Table 4 - Number of CMUs in Non-Contiguous CMUs

Supp Table 5 – CMU Representation Across Tissues

Supp Table 6 - Tissue Independent CMUs (TICMUs)

Supp Table 7 – Non-Contiguous TICMUs

Supp Table 8 – Differential Correlation
